# Identification and validation of an 18-gene signature highly-predictive of bladder cancer metastasis

**DOI:** 10.1038/s41598-017-18773-1

**Published:** 2018-01-10

**Authors:** Beihe Wang, Fangning Wan, Haoyue Sheng, Yiping Zhu, Guohai Shi, Hailiang Zhang, Bo Dai, Yijun Shen, Yao Zhu, Dingwei Ye

**Affiliations:** 1 Department of Urology, Fudan University Shanghai Cancer Center, Shanghai, 200032 P.R. China; 2 Department of Oncology, Shanghai Medical College, Fudan University, Shanghai, 200032 P.R. China

## Abstract

We found two deviant groups that were unpredictable with clinical models predicting bladder cancer metastasis. The group G consists of patients at high risk of pN+ , but they have pN0. The group P consists of patients at low risk of pN+ , but they have pN+ . We aimed to determine the genetic differences between these two groups. 1603 patients from SEER database were enrolled to build a multivariate model. This model was applied to patients from the TCGA database to distinguish groups G and P. Differentially expressed genes between the two groups were identified. RT-qPCR was used to validate the results in a cohort from FUSCC. Two deviant groups were identified both in the SEER population and the TCGA population. Expression of 183 genes was significantly different between the two groups. 18 genes achieved significant statistical power in predicting lymph node metastasis excluding these two deviant groups. The 18-gene signature outperformed 3 other bladder cancer lymph node prediction tools in 2 external GEO datasets. RT-qPCR results of our own cohort identified *NECTIN2* (P = 0.036) as the only gene that could predict metastasis. Our study showed a novel gene screening method and proposed an 18-gene signature highly predictive of bladder cancer metastasis.

## Introduction

With an incidence of approximately 7% and 4% mortality, bladder cancer has become the fourth most common cancer and the eighth most common cause of death in men^1^. In China, 80,500 new bladder cancer cases are expected with 32,900 estimated deaths for both sexes in 2015^2^. Urothelial carcinoma is the dominant histological subtype of bladder cancer, except for in certain areas in Africa and the Middle East^3^. However, despite considerable progress in management of treatment of bladder cancer, 50% of patients eventually develop metastasis^4–6^. Furthermore, bladder cancer spreads from the bladder in a predictable stepwise manner to the lymph nodes and then to visceral organs. A total of 80% of patients with pN1 disease experience recurrence of disease, while only 30% have recurrence in those with extravesical and pN0 disease^7–9^ Lymph node metastasis is a powerful predictor of cancer-specific survival^10^. Therefore, knowledge of nodal status plays a crucial rule in counseling of patients, clinical decision-making, and adjuvant chemotherapy^11,12^.

To date, many prediction models for predicting non-organ confined bladder cancer (pT3-4/N + ) have been created and properly externally validated^9,13,14^. Among these prediction tools, the nomogram developed by Karakiewicz represents the first step at defining objective, systematic, standardized, multivariate models^9^. This nomogram includes transurethral resection (TUR) stage and TUR grade to provide individual pN stage predictions. However, we found that there are two deviant groups, which are unpredictable with clinical parameters in these models. One group is at high risk of pN+, but actually has pN0 (good prognosis group, group G) and the other group is at low risk of pN+, but actually has pN+ (poor prognosis group, group P). Clinicopathological factors cannot predict outcomes in these two deviant groups. Therefore, we hypothesized that there are some genetic differences between the two populations that lead to vastly different outcomes.

In the present study, we built prediction models for pN+ disease based on the Surveillance, Epidemiology and End Results (SEER) database^15^. This study aimed to identify these two deviant groups in The Cancer Genome Atlas (TCGA) database^16^, as well as the gene signatures that are expressed differently between them. Furthermore, we validated the results in a cohort from Fudan University Shanghai Cancer Center (FUSCC).

## Results

### Construction of the pN+ disease prediction model in the SEER database

**Figure 1:**
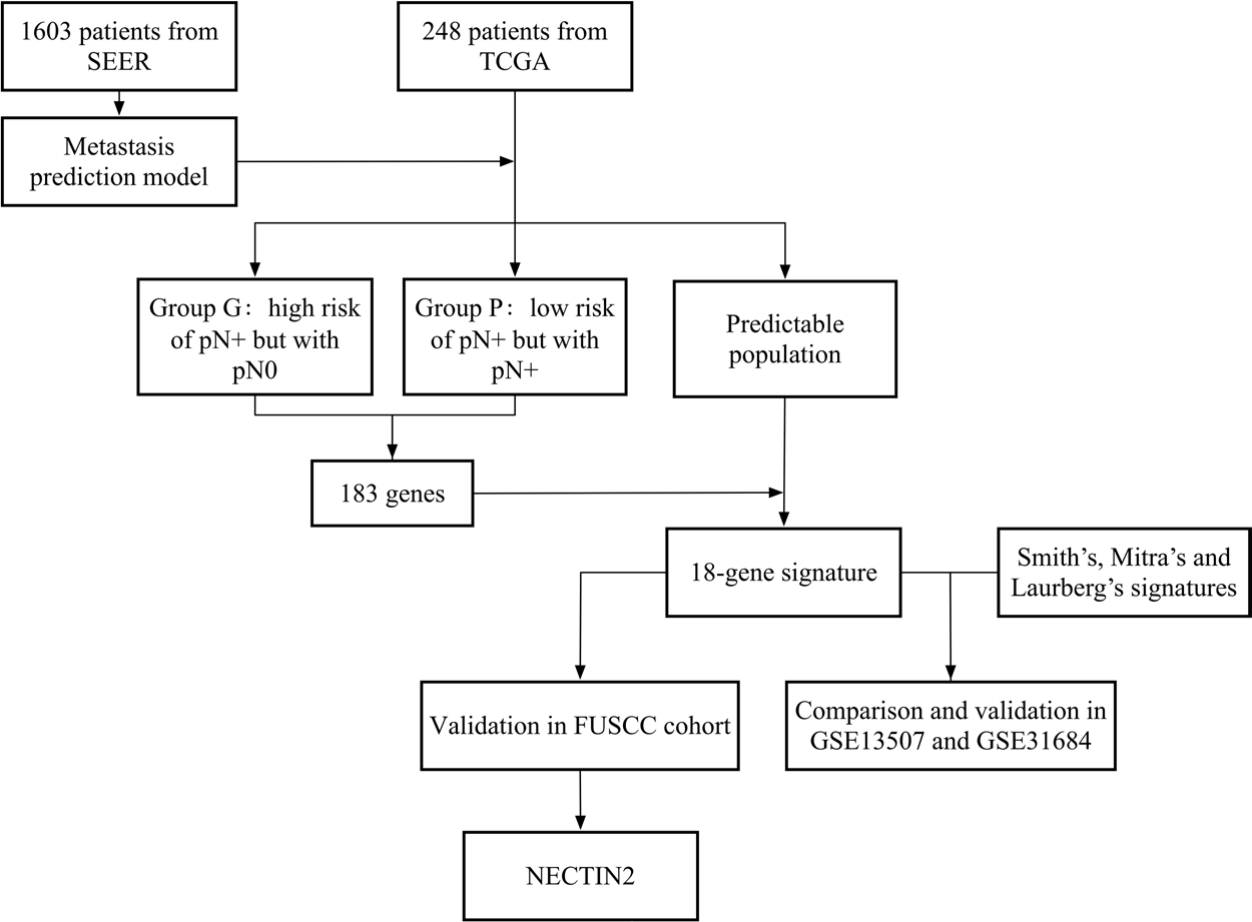
Flowchart showing the experimental design. 1603 patients from SEER database were used to build the prediction model and this model was applied in 248 patients from TCGA database to identify group G and P, as well as the predictable population. 18 of the 183 genes that differentially expressed genes between group G and P achieved statistical power for predicting metastases in the predictable population. Predictive ability of the 18-gene signature was further compared with 3 published signatures in 2 GEO datasets. The 18 genes were also validated in 130 patients from the FUSCC cohort, and *NECTIN2* was identified.

A flowchart of the experimental design and main procedures is shown in Fig. 1. The demographic characteristics of the patients in the SEER database are shown in Table 1. Among a total of 1603 patients who were available for construction of the model, 1185 (73.9%) were men and 418 (26.1) were women, with a mean age of 69 years (interquartile range: 61–76 years). The majority of patients (1107, 62.8%) had grade 4 diseases. The median number of examined regional lymph nodes was 11 (interquartile range: 6–18) and 497 (30.9%) patients harbored at least one positive lymph node.

**Table 1:** Demographic characteristics of patients in SEER, TCGA and FUSCC cohorts.

Characteristic	SEER		TCGA		FUSCC	
	NO	%	NO	%	NO	%
Age, year						
Median(IQR)	69(61–76)		70(61–77)		63(57–70)	
Gender						
Male	1185	73.9	177	71.4	108	11.1
Female	418	26.1	71	28.6	19	88.9
Grade						
Low grade	NA	NA	0	0	6	4.7
High grade	NA	NA	248	100	121	95.3
1	2	0.1	NA	NA	NA	NA
2	31	1.9	NA	NA	NA	NA
3	563	35.1	NA	NA	NA	NA
4	1007	62.8	NA	NA	NA	NA
Pathologic T stage						
2	606	37.8	66	26.6	61	48.0
3	732	45.7	139	56.0	41	32.3
4	265	16.5	43	17.3	25	18.7
Tumor size, mm						
Median(IQR)	40(25–52)		NA		NA	
Regional nodes examined count						
<15	1054	65.8	94	37.9	96	75.6
≥15	549	34.2	154	62.1	31	24.4
Lymph nodes status						
Negative	1170	69.1	152	61.3	85	66.9
Positive	496	30.9	96	38.7	42	33.1
AJCC stage						
II	456	28.5	54	21.8	55	43.3
III	603	37.6	96	38.7	40	31.5
IV	544	33.9	98	39.5	32	25.2

NA: not available, AJCC: The American Joint Committee on Cancer.

In univariate analysis of the logistic regression model with pN1 disease as the endpoint, the number of regional lymph nodes examined (HR = 1.019, 95% CI: 1.008–1.030, *P* = 0.001), tumor size (HR = 1.01, 95% CI: 1.006–1.015, *P* < 0.001), and T stage (3 vs. 2: HR = 2.844, 95% CI: 2.174–3.729, *P* < 0.001; 4 vs. 2: HR = 7.653, 95% CI: 5.507–10.636, *P* < 0.001) were identified as significant predictors, which might predict pN1 disease in patients with muscle-invasive bladder carcinoma treated with cystectomy.

**Figure 2:**
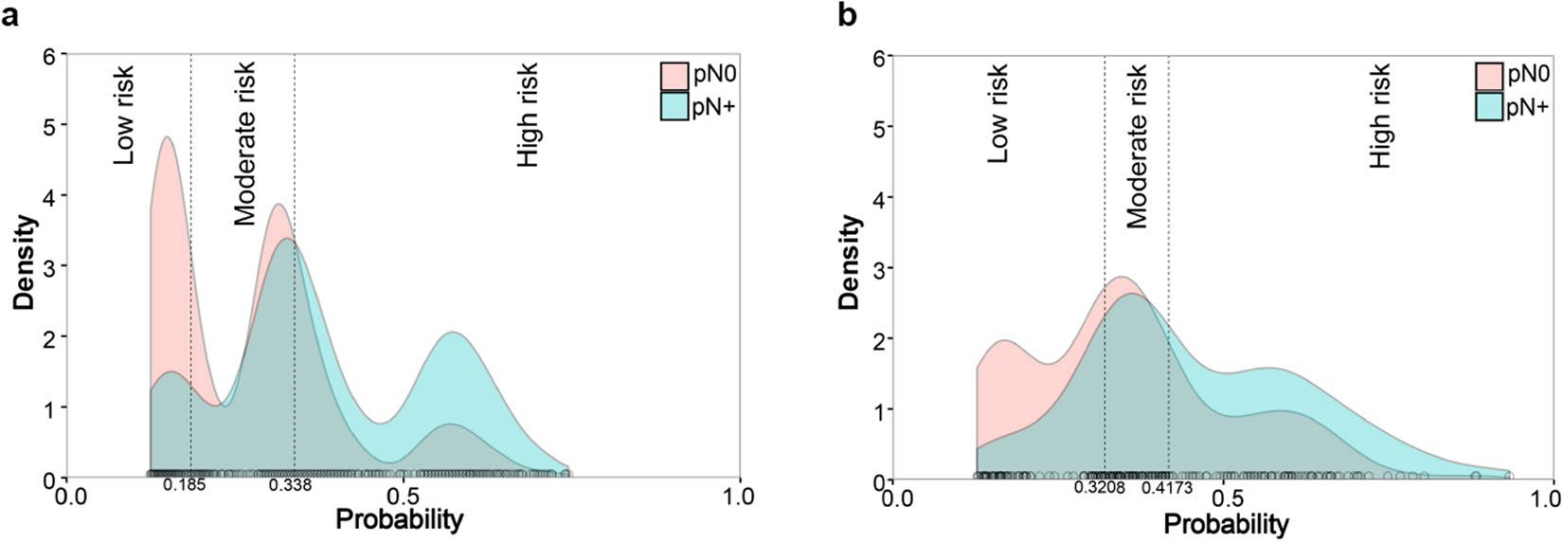
Cumulative curves showing the distribution of the two deviant groups (group G: red area in the high-risk interval; group P: blue area in the low-risk interval) in (**a**) the SEER database and (**b**) the TCGA database.

Multivariate analysis was subsequently undertaken, including all of the potential predictors that were identified in univariate analysis. With backward elimination, the number of regional lymph nodes examined (HR = 1.021, 95% CI: 1.009–1.033, *P* < 0.001) and pathologic T stage (3 vs.2: HR = 2.918, 95% CI: 2.227–3.823, *P* < 0.001; 4 vs. 2: HR = 7.721, 95% CI: 5.547–10.748, *P* < 0.001) were identified as independent predictors for pN+ disease in muscle-invasive bladder carcinoma treated with cystectomy (Table 2). The equation generated from the prediction model was as follows: probability of pN+ disease = EXP[−2.993 + 0.021 × number of lymph nodes examined + 1.025 × T stage]/(1 + EXP(−2.993 + 0.021 × number of lymph nodes examined + 1.025 × T stage)). Finally, the two unpredictable deviant groups, G (n = 256) and P (n = 76), were identified (Fig. 2a) and survival outcome was provided (Supplementary Figure 1).

**Table 2:** Univariate and multivariate analysis with pN1 as the endpoint.

Characteristic	Univariate analysis			Multivariate analysis		
	HR	95%CI	P	HR	95%CI	P
Gender						
Male	ref.					
Female	1.105	0.870–1.404	0.412			
Age	0.991	0.981–1.001	0.09			
Races						
White	ref.					
Black	1.176	0.755–1.832	0.474			
Other	0.687	0.429–1.101	0.119			
Year of diagnosis						
2000–2005	ref.					
2006–2012	0.907	0.730–1.128	0.381			
Grade						
1	ref.					
2	0.292	0.016–5.284	0.404			
3	0.478	0.030–7.680	0.602			
4	0.437	0.027–7.002	0.558			
T stage						
2	ref.			ref.		
3	2.844	2.174–3.720	<0.001	2.918	2.227–3.823	<0.001
4	7.653	5.507–10.636	<0.001	7.721	5.547–10.748	<0.001
Tumor size	1.01	1.006–1.015	<0.001			
Regional lymph nodes examined count	1.019	1.008–1.030	0.001	1.021	1.009–1.033	<0.001

### Identification of the deviant groups and genes that were expressed differently between them in the TCGA database

**Figure 3:**
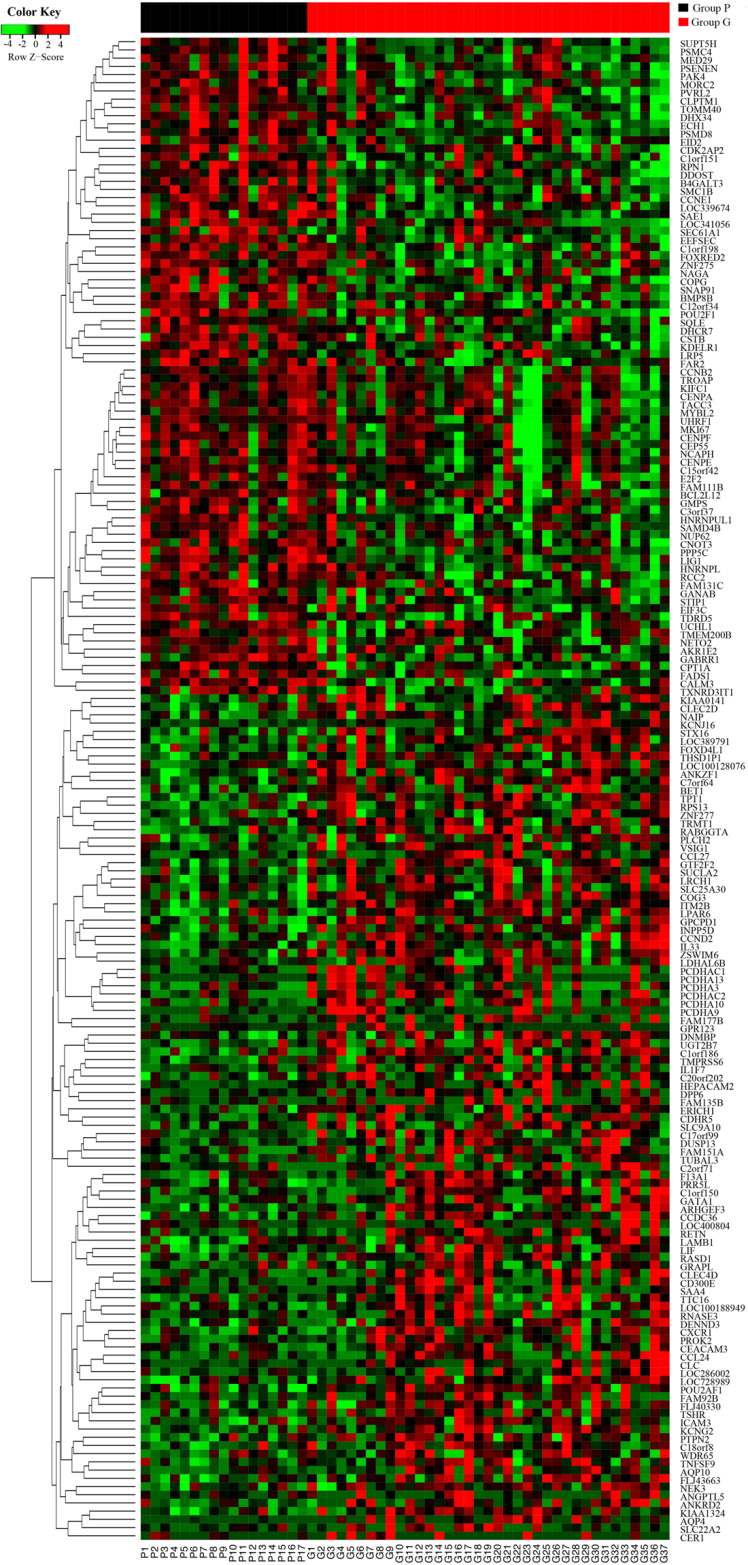
Association of gene expression with outcome in the TCGA. Heatmap showing expression of the 183 genes that were significantly (*P* < 0.05) different between group G (n = 37) and group P (n = 17). Genes are arranged by linkage distance, using unsupervised hierarchical clustering of average expression across genes as illustrated by dendrograms. Groups G and P are represented by black and red squares within the sidebar, respectively.

The baseline characteristics of patients in TCGA database are shown in Table 1. Among a total of 248 patients, the same prediction model was applied and it achieved an area under the curve (AUC) of 0.674 (95%CI: 0.612–0.732) (Supplementary Figure 2). Then, the possibility of pN + disease was generated. The cutoffs of the tertiles for the spectrum of possibilities were 0.321 and 0.417. Thirty-seven and 17 patients were identified in groups G and P respectively (Fig. 2b) and survival outcome was provided (Supplementary Figure 1). The t-test showed that 183 genes were significantly different between the two groups, as shown in the heatmap (Fig. 3 and Supplementary Table 1). To determine whether these genes could predict pN1 disease in a more common population, we excluded these two deviant groups from the total cohort. We then performed univariate logistic regression analysis with each of the 183 genes. Finally, 18 genes achieved significant statistical power (*P* < 0.05, Table 3).

**Table 3:** 18 genes that could predict lymph node metastasis in a common population.

Gene symbol	Description	P value	OR	95%CI
KDELR1	KDEL endoplasmic reticulum protein retention receptor 1	0.006	2.579	1.318–5.048
NECTIN2	nectin cell adhesion molecule 2	0.011	1.731	1.132–2.647
DHCR7	7-dehydrocholesterol reductase	0.020	1.533	1.070–2.917
C1orf198	chromosome 1 open reading frame 198	0.019	1.930	1.114–3.346
GATA1	GATA binding protein 1	0.018	1.540	1.079–2.205
TOMM40	translocase of outer mitochondrial membrane 40	0.038	0.597	0.368–0.971
TACC3	transforming acidic coiled-coil containing protein 3	0.009	0.623	0.436–0.890
EIF3C	eukaryotic translation initiation factor 3 subunit C	0.039	0.808	0.660–0.989
CENPE	centromere protein E	0.029	0.716	0.531–0.966
TRMT1	tRNA methyltransferase 1	0.021	0.572	0.356–0.919
LOC100128076	protein tyrosine phosphatase pseudogene	0.050	0.710	0.504–0.999
RCC2	regulator of chromosome condensation 2	0.043	0.526	0.282–0.981
MYBL2	MYB proto-oncogene like 2	0.040	0.777	0.061–0.989
KIFC1	kinesin family member C1	0.049	0.725	0.527–0.998
COPG	coatomer protein complex subunit gamma	0.014	2.322	1.184–4.555
FAM135B	family with sequence similarity 135 member B	0.047	1.300	1.003–1.685
TICRR	TOPBP1 interacting checkpoint and replication regulator	0.037	0.725	0.535–0.981
TROAP	trophinin associated protein	0.014	0.681	0.502–0.924

### Validating and comparing the predictive accuracy of the 18-genes signature with 3 other bladder cancer lymph node prediction tools in 2 external datasets

We performed a receiver-operating characteristic (ROC) curve analysis of the 18-gene signature as well as 3 other signatures^17–19^ in two Gene Expression Omnibus (GEO) datasets (GSE13507 and GSE31684) and compared their discrimination ability using AUC in a pairwise manner. The genes in the 3 other signatures were listed in Supplementary Table 2. Our signature achieved an AUC of 0.870 (95%CI: 0.809–0.918) and 0.816 (95%CI: 0.707–0.897) in the two datasets respectively (Supplementary Figure 3). Smith’s signature performed better than that of Mitra’s (*P* = 0.009) and Laurberg’s (*P* = 0.007) in GES 13507. However, there was no statistical difference in discrimination ability between Smith’s signature and our signature (Detail in Supplementary Figure 3 and Supplementary Table 3).

### Validating the identified genes in the FUSCC cohort

The demographic characteristics of this cohort are shown in Table 1. After the prediction model was applied, a total of 18 and 4 patients were subsequently identified in groups G and P, respectively. Validation using RT-qPCR in the FUSCC cohort showed that *NECTIN2* was the only gene that was differently expressed between the two groups (Supplementary Figure 4).

## Discussion

The present study provides important insight into the relationship between gene expression profiles and lymph node metastasis. We took advantage of the large cohort in the SEER database to construct an accurate prediction model of lymph node metastasis. Using the gene expression profile in the TCGA database, we identified the two deviant groups (G and P, Fig. 2) with a completely different genetic background, as shown by the gene expression heatmap (Fig. 3). Furthermore, we narrowed down the identified genes from 183 to 18 by examining their predictive ability in a more common population. To the best of our knowledge, for the first time, we combined the two most widely used external databases and studied the different gene expression status between extreme populations whose outcomes could not be predicted by clinicopathological factors. With external validation with RT-qPCR in a consecutive FUSCC cohort, we first give a clue that *NECTIN2* might be a trigger for metastasis in bladder cancer pending larger cohorts and basic research. Most importantly, the 18-gene signature we proposed that is highly predictive of bladder cancer metastasis outperformed three other published signatures in another two GEO datasets.

Generally, most of the studies on genetic biomarkers can be classified into one of two categories. In the first approach, some type of machine-learning algorithm is applied to the data, and thereby a panel of biomarkers is obtained. Examples of such approaches include a study carried out by Wang *et al*. In their study, 57 genes (mRNA levels) were used to classify patients with urothelial cancer at each stage into high or low risk for progression category^20^. In another study, a five-gene expression signature was developed with this type of approach to predict progression in T1G3 bladder cancer^21^.

In the second category, the authors had a particular candidate biomarker. They then separated the available patient pool into two groups according to the endpoint that they were interested in. The mean values of the candidate biomarker across each group were generated. The candidate biomarker is considered to have passed one filter for utility if there is a significant difference between these mean values. Biomarkers that are identified with this approach include *PTP*4*A3* ^22^ and *FGR3* ^23^. In such studies, there is the implicit assumption that only the putative biomarker achieves a significant variation between the groups, while other confounders may not be well balanced. Moreover, there may be more than one gene that shows a significant difference in mean values between the studied groups. Therefore, examining one or a few genes in isolation may lead to incorrect conclusions. In the present study, instead of defining criteria, identification of extreme groups was based on objective observations with the assistance of a prediction model. Furthermore, instead of choosing one or a few genes in isolation, we performed narrowing down and validation processes without prejudice.

In the development of Smith’s signature, the primary differentially expressed genes were identified simply by comparing patients pN1–3 disease and pN0 disease^19^. Although the resulted signature performed well in external validation cohorts, they didn’t take the influence of other clinicopathological factors into consideration, such as the number of lymph nodes examined. The number of lymph nodes examined has been shown a good surrogate marker of adequate sampling which is associated with the probability of getting true node status^24,25^. In the development of our 18-gene signature, by incorporating the variable ‘number of lymph nodes examined’ in the logistic regression model, the probability of a positive lymph node gets higher with the increasing number of lymph nodes examined and vice versa. To some extent, we are looking for patients with ‘extremely adequate’ sampling but have pN0 and patients with ‘extremely inadequate’ sampling but have pN+. In this manner, we have the best chance to get their true lymph node status which would minimize the influence of including the whole cohort instead of patient only with adequate sampling.

*MYBL2* and *RCC2* has been reported to play a crucial role in epithelial-to-mesenchymal transition (EMT), in which epithelial cells lose their polarity and gain migratory and invasive abilities. It has been proposed that *MYBL2* might mediate EMT and cancer cell invasion by upregulates the expression of major EMT regulator SNAIL in breast cancer^26^. *RCC2* was also reported to play a pivotal role in lung adenocarcinoma metastasis by inducing EMT via activation of MAPK-JNK signaling^27^. *NECTIN2*, which was validated in FUSCC cohort, belongs to a family consisting of four Ca^2+^-independent cell adhesion molecules (*NECTIN1* to *4*)^28^. Recent studies have shown that *NECTIN2* also contributes to tumorigenesis. Oshima *et al*. observed overexpression of *NECTIN2* in various cancer tissues and reported that *NECTIN2* is a potential target for antibody therapy^29^. However, few of these genes in bladder cancer has been previously studied. Our study only represents the first step towards defining the role of these genes in bladder cancer. Further validation, as well as more basic research, is still required.

The major limitation of this study was its retrospective design and validation was performed in a single center. We were only able to validate *NECTIN2* out of the 18 genes probably due to different populations with different genetic background and life styles. Moreover, the different platforms like RNAseq in TCGA and RT-qPCR in our study may also contribute to this problem. Considering the very limited patients in the extreme groups, we included the whole cohort instead of patients only with adequate sampling (number of examined lymph nodes ≥15) to ensure the stability of the generated signature. Despite these limitations, our study provides a useful method to screen genes between two extreme populations. This may help identify the most crucial genes in determining totally opposite outcomes. Most importantly, the proposed 18-gene signature for muscle-invasive bladder cancer patients that is highly-predictive of LN+ would help with selecting patients for neoadjuvant chemotherapy, which would benefit high-risk patients while sparing other patients’ toxic effects and delay to cystectomy.

## Patients and Methods

### Patients and samples

SEER is program that is sponsored by the National Cancer Institute and collects information on patients with cancer in the USA. This database covers approximately 26% of the American population, and it is considered representative of the USA by demographic composition, incidence of cancer, and mortality^15^. In the SEER database, we identified 2761 patients who were diagnosed with muscle-invasive urothelial cell carcinoma of bladder and were treated with radical cystectomy between 2000 and 2010. We excluded 424 patients with no examination of regional lymph nodes, 151 with missing regional lymph node data, and 583 with missing data of tumor size from the analysis. Finally, this process resulted in a cohort of 1603 patients.

The TCGA project aims to assess cancer-causing genome alternations in large cohorts of human tumors with high-throughput genomic technologies^16^. In the TCGA database, data on 408 patients who were diagnosed with muscle-invasive urothelial cell carcinoma of the bladder and were treated with radical cystectomy were collected. Samples comprised RNA sequencing data and clinical information. Of the 408 patients, we excluded 139 with missing data of the number of lymph nodes, seven with missing data of positive lymph nodes, three with missing pathological T stage, six with a history of neoadjuvant chemotherapy, and five with missing gene expression data. Finally, 248 patients were available for analysis.

In external validation of different signatures predicting lymph node metastasis, two previously published datasets (GSE13507 and GSE31684) were retrieved. A total of 164 patients from GSE13507 and 72 patients from GSE 31684 were available for analysis after exclusion of patient with missing lymph node status and other histologic type except for transitional urothelial carcinoma. 3 previously published and appropriately validated lymph node prediction tools^17–19^ were used to compare the predictive accuracy with our signature.

In validation of our own cohort, we retrospectively recruited a consecutive cohort of 130 patients with muscle-invasive urothelial cell carcinoma of the bladder between 2011 and 2015 from FUSCC. Three patients were excluded because of missing data of lymph node status. Our study was approved by the ethical committee of FUSCC and all experiments were performed in accordance with relevant guidelines and regulations. Each patient provided written informed consent before participation.

### RNA extraction and reverse transcription qPCR (RT-qPCR) analysis

For the validation cohort, 127 frozen tissue samples (100 mg) were harvested and ground into a fine powder. TRIzol® reagent (15596–026; Invitrogen, CA, USA) were used to isolate total RNA. First-strand cDNA was synthesized from total RNA with the PrimeScript RT reagent kit (K1622; Thermo Scientific, MA, USA). SYBR Green real-time PCR assays were subsequently performed using an ABI 7900HT (Applied Biosystems, CA, USA). β-actin was used as the internal reference to normalize the expression level of RNA. The primers were synthesized by Sangon (Shanghai, China). The sequences of these primers are shown in Supplementary Table 4.

### Data analysis

All statistical analyses were performed by R software (https://www.r-project.org). Categorical data are shown as frequency and percentage, and continuous data as mean and interquartile range. Univariate and multivariate logistic regression analyses were performed to assess the parameters of potential risk factors using the RMS package. The possibility of pN+ was generated in each patient and patients were divided into low-, moderate- and high-risk groups according to the tertiles of the possibilities. Groups G and P were subsequently distinguished.

Level 3 RNAseq data from bladder carcinoma samples were obtained from the TCGA data portal (https://cancergenome.nih.gov) and GEO database (https://www.ncbi.nlm.nih.gov/geo/). Gene expression levels of the two groups were compared by the t-test. The expression status of the identified genes was observed with a heatmap using the pheatmap package (https://CRAN.R-project.org/package=pheatmap). We used logistic regression analysis to identify the relationship of gene expression and lymph node metastasis. Hazard ratios (HR) and 95% confident intervals (95% CI) were calculated. Discrimination ability of classifiers were compared using AUC on ROC curves. Distributions of overall survival were estimated using Kaplan-Meier method. Two-sided *P* < 0.05 was considered as statistically significant.

### Data availability statement

The data used in this study were available publicly.

## Electronic supplementary material

Supplementary files
