# Supplementary material for: Identification and validation of an 18-gene signature highly-predictive of bladder cancer metastasis
Source: Sci Rep. 2018 Jan 10;8:374. doi: 10.1038/s41598-017-18773-1 (PMC5762631; doi:10.1038/s41598-017-18773-1)

Identification and validation of an 18-gene signature highly-predictive of bladder cancer metastasis

Beihe Wang[#1,2], Fangning Wan[#1,2], Haoyue Sheng[1,2], Yiping Zhu[1,2], Guohai Shi[1,2], Hailiang Zhang[1,2], Bo Dai[1,2], Yijun Shen [1,2*], Yao Zhu [1,2*], Dingwei Ye[1,4]*

1 Department of Urology, Fudan University Shanghai Cancer Center, Shanghai, 200032, P.R. China

2 Department of Oncology, Shanghai Medical College, Fudan University, Shanghai, 200032, P.R. China

# contributed equally to this work.

* Corresponding authors: Dr. Yijun Shen(luckysyj@gmail.com), Dr. Yao Zhu (mailzhuyao@163.com) and Dr. Dingwei Ye (dwyeli@163.com), Department of Urology, Fudan University Shanghai Cancer Center, 270 Dong'an Rd (M), Shanghai 200032, China. Tel: +86-21-64175590 Fax: +86-21-64434556

Supplementary Table 1. 183 genes identified between group G and group P in TCGA database.

Gene ID	G mean	G SD	P Mean	P SD	Absolute T value	Raw P value	Adjusted P value	FDR
CALM3	12.478	0.322	12.831	0.225	4.639	<0.001	<0.001	0.336
TPT1	15.828	0.776	15.019	0.583	4.245	<0.001	<0.001	0.864
ARHGEF3	9.037	0.666	8.420	0.460	3.946	<0.001	<0.001	1.485
GTF2F2	9.440	0.559	8.867	0.473	3.892	<0.001	<0.001	1.696
CLPTM1	11.635	0.543	12.223	0.509	3.857	0.001	0.001	1.726
LOC341056	4.205	0.594	4.850	0.569	3.817	0.001	0.001	1.715
CLEC4D	1.877	1.232	1.017	0.512	3.617	0.001	0.001	1.751
PCDHAC1	1.987	1.286	1.169	0.320	3.631	0.001	0.001	1.670
FOXRED2	8.824	0.932	9.624	0.664	3.597	0.001	0.001	1.727
HEPACAM2	1.924	1.554	0.987	0.241	3.573	0.001	0.001	1.787
EEFSEC	8.969	0.515	9.410	0.385	3.503	0.001	0.001	1.965
KDELR1	12.389	0.400	12.742	0.315	3.503	0.001	0.001	1.886
ANKZF1	9.558	0.509	9.001	0.539	3.591	0.001	0.001	1.758
LOC389791	2.868	1.068	1.895	0.885	3.509	0.001	0.001	1.644
FAM151A	2.008	1.226	1.246	0.384	3.432	0.001	0.001	1.593
PPP5C	10.326	0.275	10.718	0.398	3.675	0.001	0.001	1.515
C7orf64	7.762	0.381	7.426	0.306	3.472	0.001	0.001	1.487
ANGPTL5	1.112	0.542	0.733	0.277	3.399	0.001	0.001	1.428
C17orf99	2.277	1.339	1.364	0.634	3.396	0.001	0.001	1.366
CDHR5	2.461	1.880	1.289	0.650	3.377	0.001	0.001	1.409
TDRD5	4.031	2.344	6.451	2.359	3.508	0.001	0.001	1.348
C2orf71	1.218	0.561	0.805	0.334	3.366	0.002	0.002	1.347
KIAA1324	4.913	1.599	3.488	1.331	3.423	0.002	0.002	1.305
FAM131C	3.208	1.786	4.760	1.436	3.407	0.002	0.002	1.286
TMEM200B	6.211	2.069	7.641	1.075	3.337	0.002	0.002	1.251
POU2AF1	6.368	1.413	4.305	2.160	3.601	0.002	0.002	1.208
CENPF	10.384	0.986	11.181	0.711	3.366	0.002	0.002	1.202
DDOST	12.799	0.358	13.163	0.360	3.458	0.002	0.002	1.168
HNRNPL	12.618	0.303	12.946	0.329	3.475	0.002	0.002	1.150
RPN1	12.926	0.405	13.296	0.356	3.399	0.002	0.002	1.126
C1orf150	1.692	0.980	1.076	0.383	3.311	0.002	0.002	1.100
TUBAL3	2.167	1.431	1.243	0.620	3.310	0.002	0.002	1.068
PVRL2	11.457	0.676	12.036	0.540	3.367	0.002	0.002	1.057
C12orf34	5.358	1.143	6.367	0.964	3.364	0.002	0.002	1.076
IL33	7.721	2.543	5.724	1.781	3.321	0.002	0.002	1.047
DHCR7	10.271	0.854	11.075	0.791	3.380	0.002	0.002	1.042
ECH1	11.676	0.650	12.400	0.751	3.428	0.002	0.002	1.062
GPCPD1	9.421	0.774	8.766	0.621	3.320	0.002	0.002	1.051
C1orf198	10.357	0.590	10.834	0.441	3.306	0.002	0.002	1.030
KCNG2	1.725	1.032	1.081	0.422	3.247	0.002	0.002	1.035

SMC1B	3.469	2.029	5.607	2.210	3.386	0.002	0.002	1.034
UGT2B7	4.015	2.762	2.072	1.619	3.237	0.002	0.002	1.048
SLC9A10	1.535	0.791	1.023	0.379	3.216	0.002	0.002	1.055
GATA1	1.477	0.852	0.952	0.353	3.194	0.002	0.002	1.097
AKR1E2	5.150	1.912	6.307	0.756	3.181	0.003	0.003	1.116
RABGGTA	9.485	0.527	9.081	0.379	3.198	0.003	0.003	1.148
ANKRD2	2.930	1.841	1.703	1.022	3.136	0.003	0.003	1.238
SAE1	11.437	0.311	11.818	0.424	3.313	0.003	0.003	1.222
LOC339674	2.155	1.047	3.153	1.055	3.235	0.003	0.003	1.215
FLJ40330	3.436	1.455	2.226	1.226	3.170	0.003	0.003	1.249
NETO2	7.996	1.567	9.120	1.036	3.123	0.003	0.003	1.234
TOMM40	10.901	0.567	11.522	0.686	3.257	0.003	0.003	1.211
GABRR1	2.312	1.829	4.488	2.440	3.278	0.003	0.003	1.209
CCL24	1.816	1.185	1.042	0.645	3.097	0.003	0.003	1.197
ERICH1	8.197	0.531	7.722	0.499	3.180	0.003	0.003	1.197
TACC3	10.503	0.876	11.113	0.552	3.103	0.003	0.003	1.178
RPS13	13.029	0.732	12.467	0.551	3.126	0.003	0.003	1.165
ZNF275	8.533	0.561	8.932	0.372	3.097	0.003	0.003	1.170
GPR123	1.219	0.731	0.799	0.266	3.077	0.003	0.003	1.160
AQP10	1.927	1.542	1.070	0.480	3.072	0.004	0.004	1.187
EID2	8.053	0.460	8.535	0.540	3.190	0.004	0.004	1.187
CCDC36	2.355	1.036	1.617	0.695	3.076	0.004	0.004	1.173
NUP62	10.932	0.388	11.364	0.491	3.201	0.004	0.004	1.190
FAR2	6.512	1.604	7.962	1.569	3.132	0.004	0.004	1.191
PCDHA10	2.273	1.398	1.350	0.820	3.037	0.004	0.004	1.199
DHX34	9.416	0.484	9.794	0.386	3.075	0.004	0.004	1.193
SUCLA2	9.387	0.669	8.895	0.485	3.058	0.004	0.004	1.183
CENPA	7.389	1.042	8.104	0.666	3.037	0.004	0.004	1.170
LIG1	10.092	0.540	10.562	0.506	3.101	0.004	0.004	1.154
C20orf202	2.669	0.912	1.934	0.771	3.066	0.004	0.004	1.186
EIF3C	7.688	1.204	8.578	0.891	3.036	0.004	0.004	1.184
FADS1	9.435	1.128	10.377	1.005	3.073	0.004	0.004	1.169
HNRNPUL1	12.474	0.530	12.941	0.511	3.084	0.004	0.004	1.161
LOC400804	1.080	0.489	0.790	0.227	2.987	0.004	0.004	1.183
CENPE	8.558	0.911	9.222	0.664	3.018	0.004	0.004	1.177
IL1F7	1.587	1.075	0.984	0.406	2.983	0.004	0.004	1.173
C1orf186	4.516	2.402	2.587	2.053	3.036	0.004	0.004	1.167
CLC	1.675	1.497	0.919	0.199	3.018	0.005	0.005	1.177
TRMT1	9.972	0.589	9.528	0.460	3.009	0.005	0.005	1.175
CNOT3	10.492	0.426	10.858	0.403	3.044	0.005	0.005	1.175
BET1	9.022	0.453	8.685	0.349	2.997	0.005	0.005	1.184
RNASE3	1.285	0.832	0.846	0.238	2.962	0.005	0.005	1.193
TSHR	1.595	0.978	1.050	0.375	2.948	0.005	0.005	1.186
VSIG1	4.560	2.377	3.091	1.279	2.943	0.005	0.005	1.186

CXCR1	2.949	1.637	1.758	1.227	2.967	0.005	0.005	1.206
SQLE	10.537	0.969	11.196	0.648	2.944	0.005	0.005	1.218
SAMD4B	10.538	0.641	11.049	0.558	2.980	0.005	0.005	1.215
UHRF1	8.826	0.962	9.364	0.389	2.919	0.005	0.005	1.204
CCNE1	8.379	1.150	9.223	0.886	2.949	0.005	0.005	1.223
RASD1	7.101	1.746	5.872	1.255	2.937	0.005	0.005	1.210
PSMC4	11.155	0.576	11.698	0.633	3.010	0.005	0.005	1.238
B4GALT3	10.569	0.619	11.230	0.793	3.038	0.006	0.006	1.242
LOC100128076	1.650	1.044	1.043	0.498	2.895	0.006	0.006	1.205
RCC2	12.408	0.481	12.756	0.366	2.929	0.006	0.006	1.196
UCHL1	6.921	2.338	8.499	1.575	2.912	0.006	0.006	1.191
COG3	9.407	0.577	8.858	0.645	2.998	0.006	0.006	1.182
SLC25A30	7.875	0.879	7.263	0.629	2.913	0.006	0.006	1.184
GANAB	13.163	0.408	13.470	0.332	2.930	0.006	0.006	1.185
PSMD8	11.664	0.424	12.297	0.796	3.087	0.006	0.006	1.182
ZNF277	8.701	0.614	8.308	0.376	2.887	0.006	0.006	1.179
PCDHA3	2.754	1.693	1.689	1.002	2.882	0.006	0.006	1.176
LPAR6	9.208	1.169	8.060	1.373	2.986	0.006	0.006	1.174
MORC2	10.099	0.446	10.547	0.538	2.989	0.006	0.006	1.181
SLC22A2	1.310	0.867	0.853	0.293	2.867	0.006	0.006	1.180
FOXD4L1	2.914	1.156	2.129	0.804	2.885	0.006	0.006	1.170
FAM92B	1.416	0.887	0.959	0.264	2.869	0.006	0.006	1.171
LOC728989	1.718	0.726	1.195	0.560	2.892	0.006	0.006	1.174
TTC16	1.777	0.955	1.177	0.571	2.861	0.006	0.006	1.163
DUSP13	2.252	1.606	1.342	0.738	2.852	0.006	0.006	1.158
MYBL2	10.469	1.240	11.251	0.751	2.859	0.006	0.006	1.157
SAA4	1.963	1.788	1.064	0.452	2.865	0.006	0.006	1.156
PAK4	10.907	0.791	11.449	0.566	2.870	0.006	0.006	1.152
LOC286002	1.305	0.856	0.861	0.277	2.849	0.006	0.006	1.151
C3orf37	10.217	0.416	10.617	0.483	2.952	0.006	0.006	1.144
LRCH1	8.739	0.676	8.228	0.568	2.889	0.007	0.007	1.142
BCL2L12	9.139	0.561	9.516	0.389	2.860	0.007	0.007	1.134
POU2F1	6.255	1.120	7.222	1.133	2.922	0.007	0.007	1.130
DPP6	1.491	1.197	0.905	0.230	2.865	0.007	0.007	1.121
PCDHAC2	2.410	1.751	1.399	0.870	2.833	0.007	0.007	1.118
CDK2AP2	10.572	0.627	11.020	0.483	2.870	0.007	0.007	1.110
DENND3	8.173	0.951	7.509	0.711	2.855	0.007	0.007	1.133
DNMBP	9.613	0.904	8.982	0.674	2.855	0.007	0.007	1.126
BMP8B	6.104	1.570	7.476	1.632	2.903	0.007	0.007	1.127
LDHAL6B	1.384	0.757	0.942	0.394	2.818	0.007	0.007	1.119
ZSWIM6	8.219	0.726	7.684	0.595	2.860	0.007	0.007	1.120
NAGA	10.228	0.537	10.610	0.415	2.850	0.007	0.007	1.114
TXNRD3IT1	7.536	0.531	7.982	0.526	2.884	0.007	0.007	1.127
KIFC1	9.525	0.994	10.126	0.568	2.809	0.007	0.007	1.124

E2F2	6.965	1.112	7.789	0.926	2.843	0.007	0.007	1.147
FAM177B	1.262	0.718	0.872	0.308	2.789	0.007	0.007	1.153
AQP4	1.502	1.188	0.937	0.181	2.825	0.007	0.007	1.145
PSENEN	10.577	0.740	11.106	0.584	2.829	0.007	0.007	1.137
STIP1	12.037	0.511	12.363	0.331	2.799	0.008	0.008	1.144
FLJ43663	6.461	0.968	5.741	0.817	2.831	0.008	0.008	1.139
PTPN2	9.606	0.536	9.214	0.441	2.825	0.008	0.008	1.135
CCND2	9.559	1.751	8.406	1.217	2.798	0.008	0.008	1.139
LIF	8.466	1.685	7.252	1.363	2.816	0.008	0.008	1.133
FAM111B	7.606	1.126	8.409	0.894	2.815	0.008	0.008	1.127
CEACAM3	1.798	1.167	1.172	0.494	2.769	0.008	0.008	1.137
C18orf8	9.054	0.651	8.614	0.477	2.794	0.008	0.008	1.138
ICAM3	8.365	0.651	7.855	0.598	2.829	0.008	0.008	1.131
NCAPH	8.575	0.991	9.421	1.025	2.847	0.008	0.008	1.125
TNFSF9	5.438	1.914	4.187	1.319	2.787	0.008	0.008	1.118
CER1	1.092	0.353	0.874	0.220	2.773	0.008	0.008	1.125
NAIP	5.083	1.209	4.238	0.942	2.792	0.008	0.008	1.129
CD300E	1.681	1.142	1.087	0.435	2.758	0.008	0.008	1.123
CPT1A	9.707	1.318	10.658	1.079	2.799	0.008	0.008	1.115
STX16	10.930	0.439	10.583	0.412	2.818	0.008	0.008	1.109
KIAA0141	9.757	0.523	9.371	0.444	2.798	0.008	0.008	1.115
SEC61A1	13.131	0.326	13.476	0.442	2.880	0.008	0.008	1.114
MKI67	10.989	1.035	11.691	0.772	2.777	0.008	0.008	1.115
RETN	1.797	1.232	1.099	0.632	2.746	0.008	0.008	1.109
THSD1P1	6.920	0.563	6.411	0.633	2.837	0.008	0.008	1.110
KCNJ16	1.387	1.169	0.831	0.242	2.766	0.008	0.008	1.104
C1orf151	10.433	0.379	10.747	0.380	2.815	0.008	0.008	1.097
CEP55	9.095	0.954	9.826	0.862	2.798	0.008	0.008	1.093
WDR65	1.918	0.876	1.276	0.743	2.784	0.008	0.008	1.097
SNAP91	2.262	1.720	4.407	2.828	2.892	0.009	0.009	1.120
LOC100188949	2.556	1.284	1.713	0.917	2.750	0.009	0.009	1.116
CCNB2	9.453	0.979	10.010	0.522	2.721	0.009	0.009	1.131
CSTB	13.113	1.060	13.893	0.913	2.768	0.009	0.009	1.127
TMPRSS6	2.920	1.210	2.082	0.950	2.751	0.009	0.009	1.123
COPG	12.110	0.399	12.474	0.459	2.815	0.009	0.009	1.119
MED29	10.256	0.473	10.704	0.571	2.819	0.009	0.009	1.124
INPP5D	9.433	1.295	8.244	1.508	2.807	0.009	0.009	1.126
CLEC2D	9.136	0.453	8.794	0.408	2.763	0.009	0.009	1.122
PLCH2	8.589	2.060	6.997	1.918	2.767	0.009	0.009	1.118
PCDHA13	1.850	1.738	1.047	0.284	2.730	0.009	0.009	1.132
FAM135B	1.211	0.644	0.880	0.257	2.699	0.009	0.009	1.130
C15orf42	7.684	1.030	8.292	0.612	2.699	0.010	0.010	1.142
F13A1	8.182	1.701	7.078	1.218	2.715	0.010	0.010	1.137
SUPT5H	11.654	0.500	12.049	0.483	2.756	0.010	0.010	1.130

PROK2	1.539	1.082	1.005	0.359	2.698	0.010	0.010	1.126
ITM2B	13.737	0.652	12.866	1.175	2.861	0.010	0.010	1.127
TROAP	8.833	1.136	9.434	0.509	2.688	0.010	0.010	1.122
PCDHA9	1.356	0.774	0.986	0.209	2.702	0.010	0.010	1.118
GMPS	10.331	0.564	10.766	0.529	2.745	0.010	0.010	1.115
LAMB1	12.315	0.631	11.833	0.584	2.743	0.010	0.010	1.113
GRAPL	1.520	0.779	0.999	0.590	2.713	0.010	0.010	1.109
PRR5L	7.207	1.424	6.142	1.285	2.733	0.010	0.010	1.116
LRP5	10.925	0.604	11.444	0.656	2.767	0.010	0.010	1.111
NEK3	7.308	0.751	6.696	0.762	2.752	0.010	0.010	1.109
CCL27	1.360	0.935	0.921	0.217	2.696	0.010	0.010	1.106

Supplementary Table 2. List of genes constituting the signatures in external validation with GSE13507 and GSE31684 datasets.

Smith et al.	Mitra et al.	Laurberg et al
TOX3	FOXO6	COL6A2
SLC11A2	HSD17B7	LMCD1
FAM36A	ARID4B	FZD1
LIMCH1	ENAH	MITF
RAB15	MAP4K3	EDNRA
AVL9	MARCH7	EBF1
PCMTD2	MECOM	TPST1
PTHLH	LRBA	AEBP1
DPP4	MUT	PALLD
PCDHGA10	CRCP	GEM
MT1E	SYPL1	PXDN
MAP4K4	ARFGEF1	KITLG
SLC16A1	EHF	
BST2	METTL7A	
MMP14	PPP1R12A	
IFI27		
NCLN		
HLA-G		
RRBP1		
ICAM1		

Supplementary Table 3. Comparison of the receiver operating characteristic (ROC) curves of the 4 signatures in a pairwise manner in GSE13507 and GSE31684 datasets.

	**Wang et al.**	**Smith et al.**	**Mitra et al.**	**Laurberg et al.**
**GSE13507**	*P* value	*P* value	*P* value	*P* value
**Wang et al.**	-	0.16	0.08	0.09
**Smith et al.**	0.16	-	0.009	0.007
**Mitra et al.**	0.08	0.009	-	0.82
**Laurberg et al.**	0.09	0.007	0.82	-
**GSE31684**	*P* value	*P* value	*P* value	*P* value
**Wang et al.**	-	0.8	0.91	0.98
**Smith et al.**	0.8	-	0.7	0.75
**Mitra et al.**	0.91	0.7	-	0.93
**Laurberg et al.**	0.09	0.75	0.93	-

Supplementary Table 4. Sequences of the primers.

Gene ID	Sequences
TROAP	forward primer: 5'- CCTCCGGGGTGTATCTCCTAC-3'
	reverse primer: 5'- ACGGCGCACGATGTAACAG -3'
KDELR1	forward primer: 5'- TCAAAGCTACTTACGATGGGAAC-3'
	reverse primer: 5'- ATTGACCAGGAACGCCAGAAT-3'
PVRL2	forward primer: 5'- GGATGTGCGAGTTCAAGTGCT -3'
	reverse primer: 5'- TGGGACCCATCTTAGGGTGG-3'
C1orf198	forward primer: 5'- GCTGACCCCTAGCCAGATCA-3'
	reverse primer: 5'- CTCTCACGTTCGGTGCTCAC-3'
GATA1	forward primer: 5'- TGCGGCCTCTATCACAAGATG-3'
	reverse primer: 5'- CTGCCCGTTTACTGACAATCA-3'
TOMM40	forward primer: 5'- AAGCTCACAGTCAACAAAGGG-3'
	reverse primer: 5'- GGTAGTTGGACTCCCCGATTG-3'
TACC3	forward primer: 5'- TCGCCACCAGAAGTTACCG-3'
	reverse primer: 5'- TCCCGCAGAGGTGTCTGAAA-3'
EIF3C	forward primer: 5'- TACCAAGAGAGTTGTCCGCAG-3'
	reverse primer: 5'- GGTGACATCACGAATCTTCATGG-3'
CENPE	forward primer: 5'- GATGACCTAGCAACTACACAGTC-3'
	reverse primer: 5'- AAAGCACCCAAACTCGAATCA-3'
TRMT1	forward primer: 5'- GGCTGCCAAAATCGCCTTTC-3'
	reverse primer: 5'- AATGCGAGCAAACTCGGTGAT-3'
RCC2	forward primer: 5'- AAGGAGCGCGTCAAACTTGAA-3'
	reverse primer: 5'- GCTTGCTGTTTAGGCACTTCTT-3'
MYBL2	forward primer: 5'- CCGGAGCAGAGGGATAGCA-3'
	reverse primer: 5'- CAGTGCGGTTAGGGAAGTGG-3'
KIFC1	forward primer: 5'- GTGTTCCACAGCTATTGCCAC-3'
	reverse primer: 5'- ACACCTGATGTGCCAGACTTC-3'
FAM135B	forward primer: 5'- CAAGGAACGGTTGAGTTTTCGG-3'
	reverse primer: 5'- CTCGGATCTGGTAATACCCTCTC-3'
C15orf42	forward primer: 5'- TGCTGTCACAAAGTAATGCTGC-3'
	reverse primer: 5'- GCAACTCAGATAGGTGAGGAGG-3'
DHCR7	forward primer: 5'- GCAGGGGTTGTGAACAAGTAT-3'
	reverse primer: 5'- GAGACGGCATAGCCAAGGAT-3'
LOC100128076	forward primer: 5'- GTCACAGACATGGTGACGGT-3'
	reverse primer: 5'- TTCTTGTTGTTGCCCCGAGA-3'
COPG	forward primer: 5'- CGAGTGGTCTTGGAGCATGA-3'
	reverse primer: 5'- GTGGCTCGGTCCCTTACTTC-3'
beta-ACTIN	forward primer: 5'- CATGTACGTTGCTATCCAGGC-3'
	reverse primer: 5'- CTCCTTAATGTCACGCACGAT-3'

Supplementary Figure 1. Kaplan-Meier analysis of overall survival between the two deviant groups. Overall survival of Group G and Group P in (a) SEER database and (b) TCGA database.

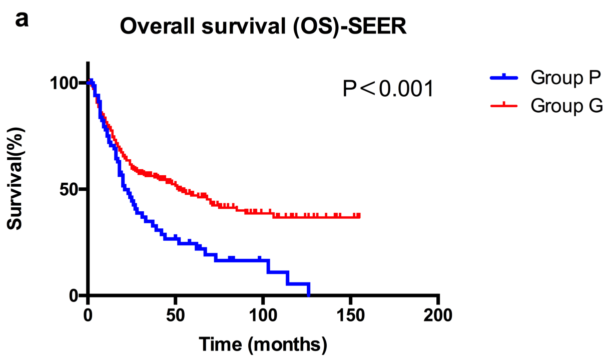

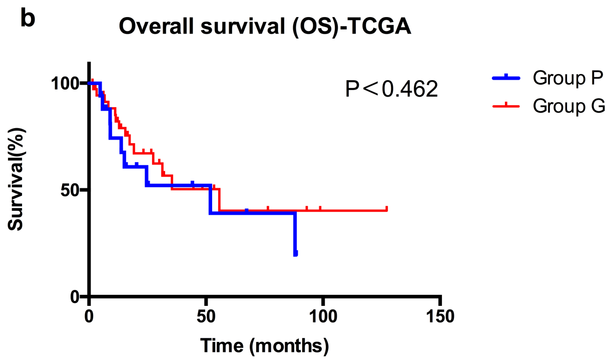

Supplementary Figure 2. Receiver operating characteristic (ROC) curve analysis of the clinical lymph node prediction model in SEER and TCGA populations.

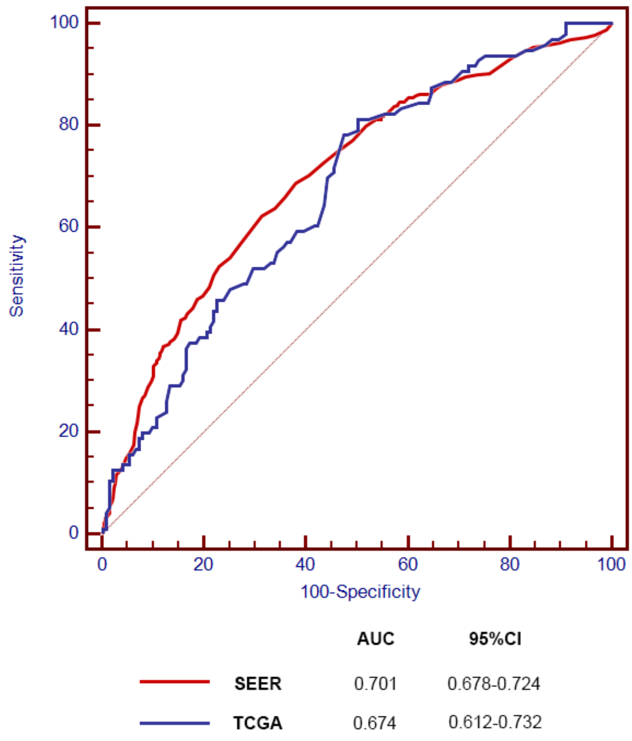

Supplementary Figure 3. Receiver operating characteristic (ROC) curve analysis of our 18-gene signature as well as 3 other lymph node prediction signatures in two external datasets. Comparison of AUC of the 4 signatures in (a) GSE 13507 dataset and (b) GSE 31684 dataset.

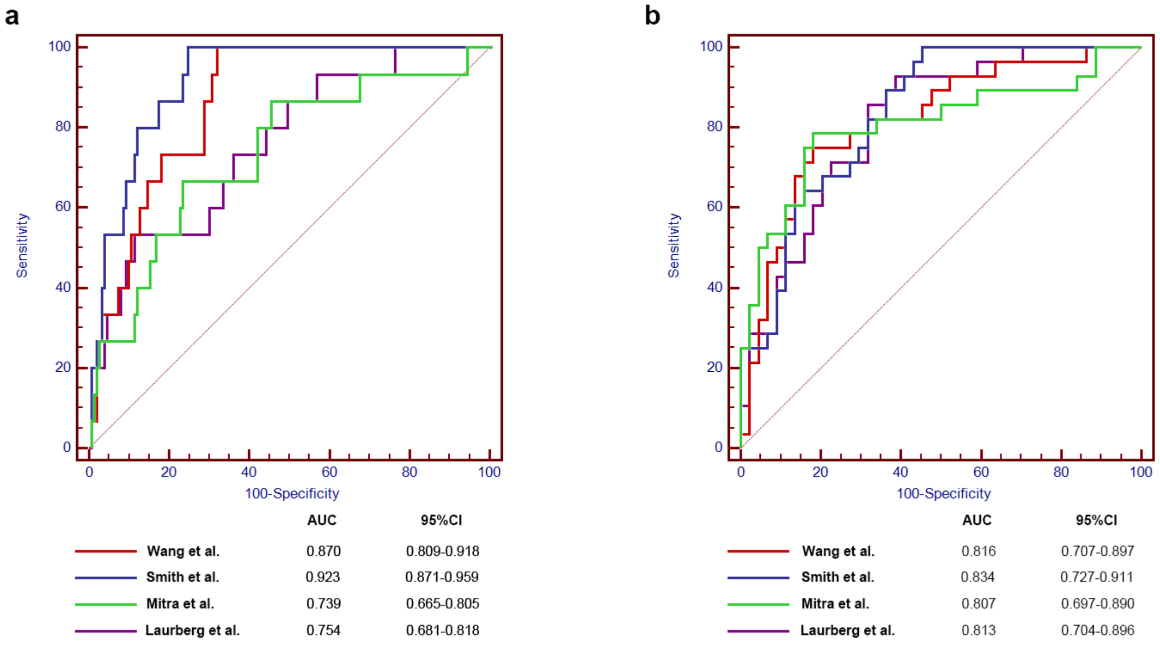

Supplementary Figure 4. mRNA expressions of the 18 genes between the two deviant groups. Validation using RT-qPCR in the FUSCC cohort showed that *NECTIN2* was the only gene that was differently expressed between the two groups.

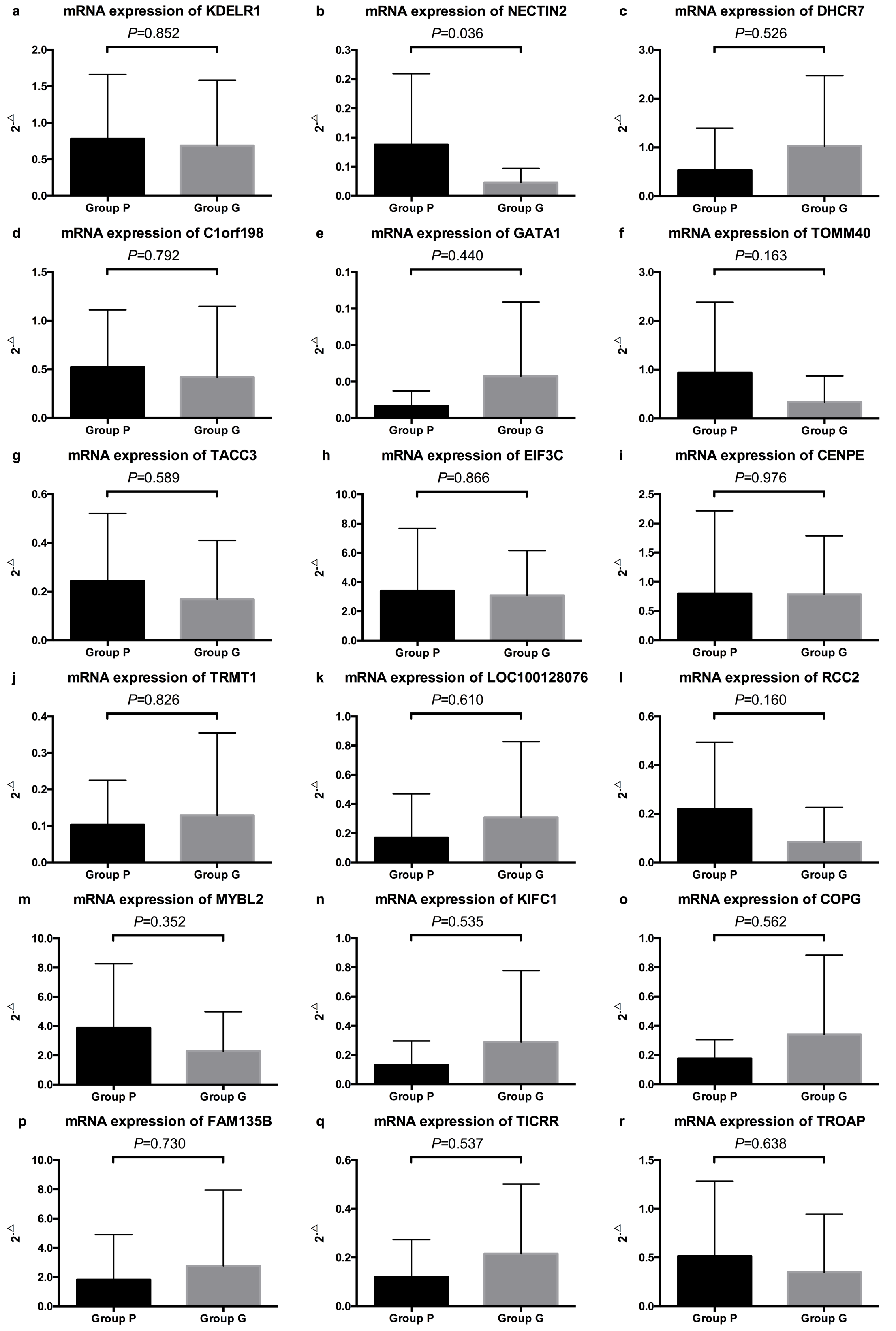

Supplement: Supplementary file 1 — Supplementary files [file 41598_2017_18773_MOESM1_ESM.pdf]
